# First person – Prachi Joshi, Arnav Saha and Radhika Malaviya

**DOI:** 10.1242/bio.062647

**Published:** 2026-05-19

## Abstract

First Person is a series of interviews with the first authors of a selection of papers published in Biology Open, helping researchers promote themselves alongside their papers. Prachi Joshi, Arnav Saha and Radhika Malaviya are co-first authors on ‘ AXL receptor tyrosine kinase regulates Golgi organization and function via an adhesion-Arf1 signalling axis in breast and lung cancer cell lines’, published in BiO. Prachi conducted the research described in this article while a PhD student in Professor Nagaraj Balasubramanian's lab at Indian Institute of Science Education and Research (IISER) Pune, Pune, India. She is now a postdoctoral associate in the lab of Professor Martin Schwartz at Yale University Cardiovascular Research Center, New Haven, CT, USA, investigating the mechanisms by which cells respond to extracellular mechanical cues and how these signals regulate cell function and contribute to disease. Arnav is a PhD student in the lab of Professor Nagaraj Balasubramanian at IISER Pune, investigating how cell-extracellular (ECM) adhesion, signal transduction and organelle mechanobiology integrate to regulate cancer mechanobiology and cellular behaviour. Radhika is a PhD student in the same lab, investigating the effects of cell-ECM adhesion and matrix stiffness on organelle structure and function.

**Figure:**
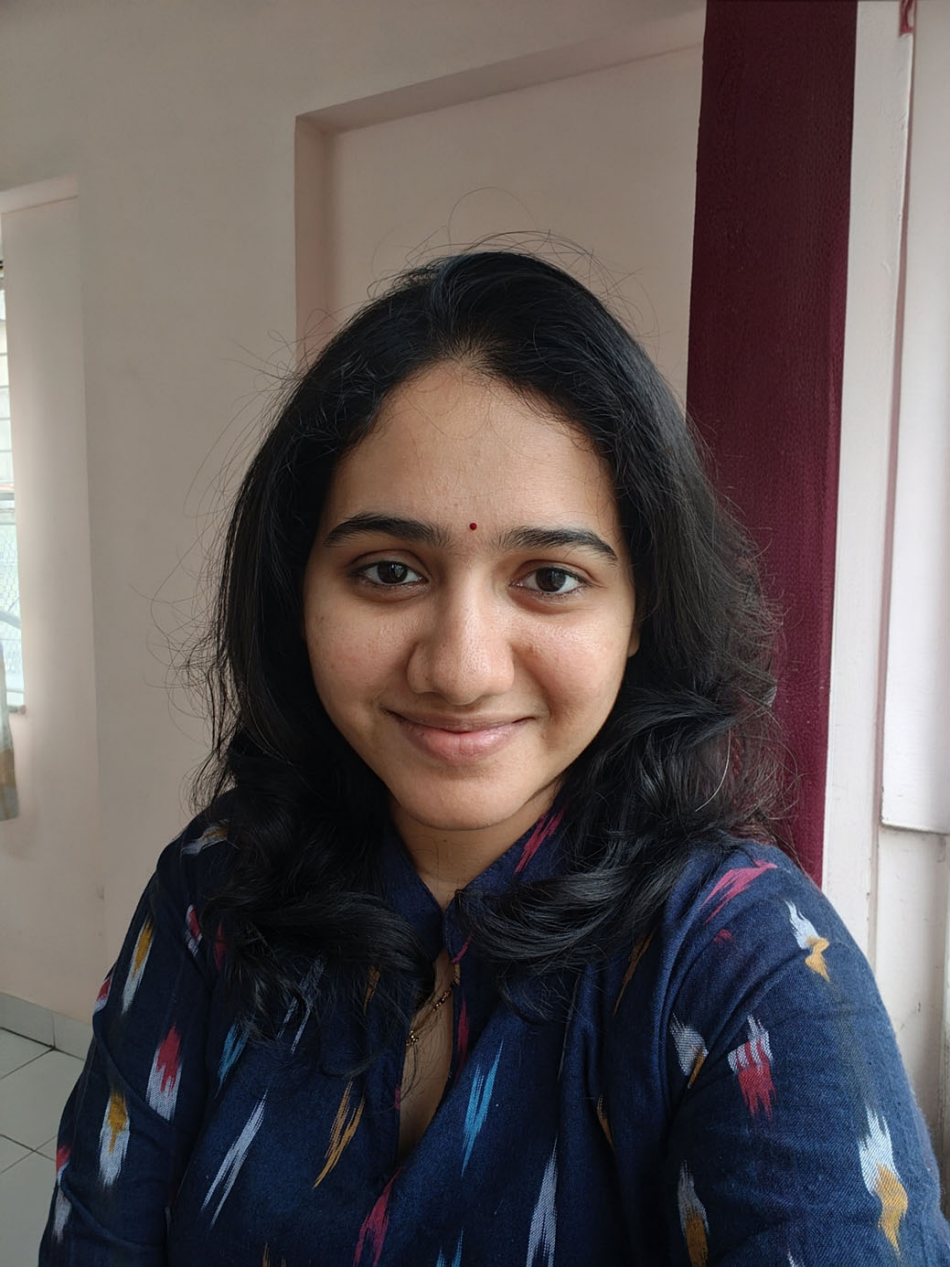
Prachi Joshi

**Describe your scientific journey and your current research focus**

**P.J.:** I completed my undergraduate studies in biotechnology, where I was first introduced to research. During my PhD, I investigated how cell-matrix adhesion regulates Golgi organization and function in cancer, leading to identification of a novel role for AXL at the Golgi. I am currently a postdoctoral associate investigating the mechanobiology of endothelial cell membranes in the context of vascular diseases.

**A.S.:** My scientific journey has progressed from early training in molecular biology and biotechnology to a PhD at IISER Pune, where I investigated how cell-ECM adhesion and mechanosensitive signalling regulate Golgi organization and functions in breast cancer cells, with a focus on the AXL-Arf1 signalling axis.

**R.M.:** I have received academic and research training in cell biology during the integrated Master's and PhD program at IISER Pune. My PhD thesis work focuses on how Golgi organization and function are regulated by cell-ECM adhesion and matrix stiffness, with a particular emphasis on how this regulation differs between normal and cancer cells.

**Who or what inspired you to become a scientist?**

**P.J.:** I have always enjoyed learning biology, but my interest in exploring it through research was truly shaped through my coursework in biotechnology, with guidance of excellent mentors. What continues to motivate me is the intellectual and personal growth that research fosters and the opportunity to contribute meaningfully beyond oneself.

**A.S.:** I was drawn to science by the intellectual freedom it offers – the ability to ask questions, explore ideas across disciplines and continuously learn. Along the way, moments where experiments challenged my assumptions have been especially formative, pushing me to think more critically and grow. This journey has also been shaped by inspiring mentors and excellent teachers – particularly during my Master's training – who played a significant role in nurturing my curiosity and approach to science.

**R.M.:** I have always been drawn to things that will increase my understanding of the world. Science and research were the obvious choice. My high-school teachers have played an instrumental role in giving me a new perspective and appreciation for biology.

**How would you explain the main finding of your paper?**

**A.S.:** Our study shows that the way cancer cells attach to their surroundings influences the structure and function of the Golgi, a key compartment that processes and distributes molecules inside the cell. We identified a signalling pathway involving two key proteins, AXL and Arf1, that links these external attachment cues to the cell's internal organization. When this AXL-Arf1 axis is disrupted, the Golgi becomes disorganized and functions poorly, leading to changes in how cells function. This highlights how signals from a cell's environment can directly control its internal machinery and behaviour.

Our study shows that the way cancer cells attach to their surroundings influences the structure and function of the Golgi

**What are the potential implications of this finding for your field of research?**

**P.J.:** Despite advances in cancer research, many underlying mechanisms remain unclear. While AXL has frequently been targeted in clinical trials, how these therapies work at a cellular level is not fully understood. Similarly, changes in Golgi organization and function are common in cancer, but the reasons behind them are still being uncovered. Our findings propose a new mechanistic link between these processes, helping to better understand how cancer cells function and potentially informing future therapeutic strategies.

**A.S.:** The identification of the AXL-Arf1 pathway as a regulator of Golgi organization highlights a previously unrecognized link between a cell's external environment and its internal organelles. Beyond validating our initial screening approach, this finding suggests that similar strategies can uncover new principles governing organelle organization. Importantly, it opens up new directions for understanding how disruptions in such pathways contribute to diseases like cancer, and points to potential targets for therapeutic intervention.

**R.M.:** The slight variations in this signalling axis between breast and lung cancer cells also highlight the inherent heterogeneity present between different cancers.

**Figure:**
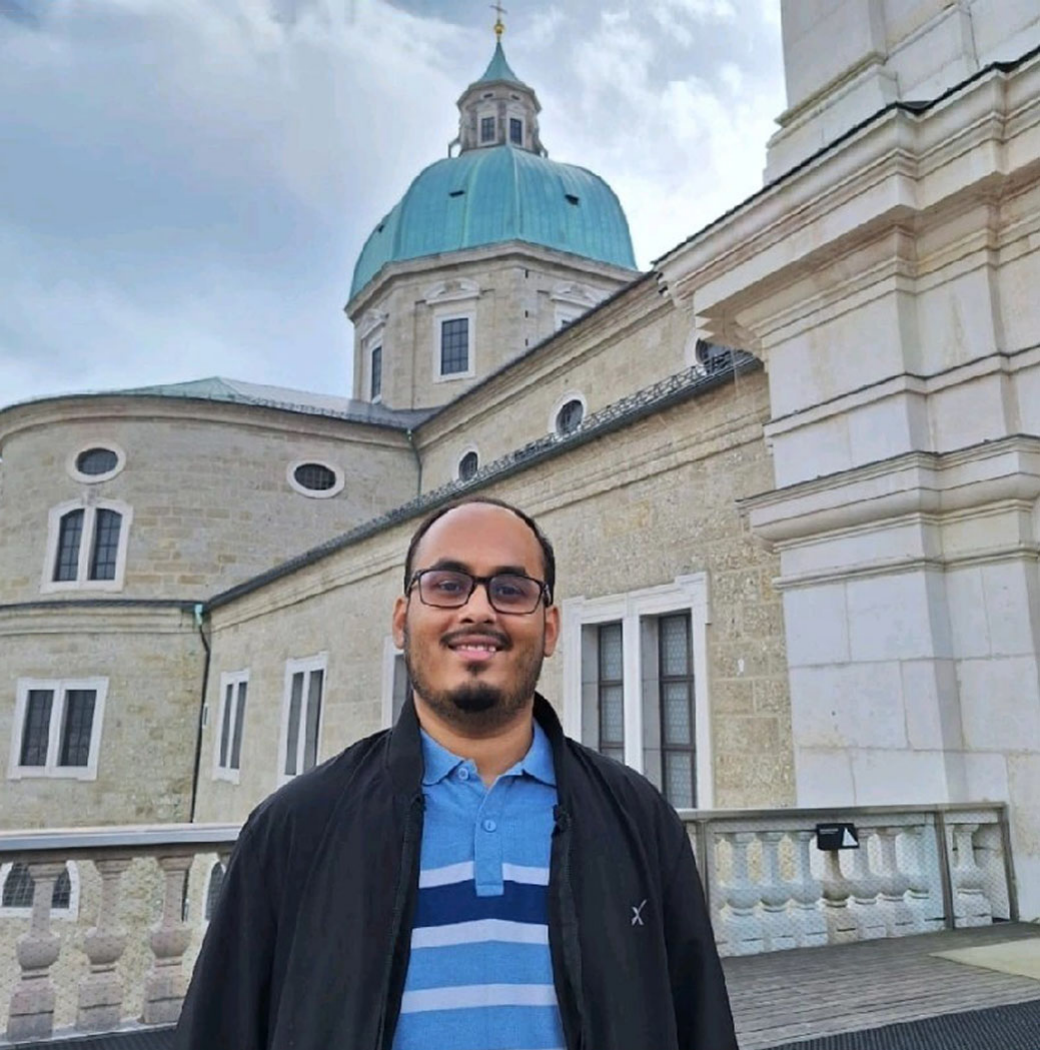
Arnav Saha

**Figure:**
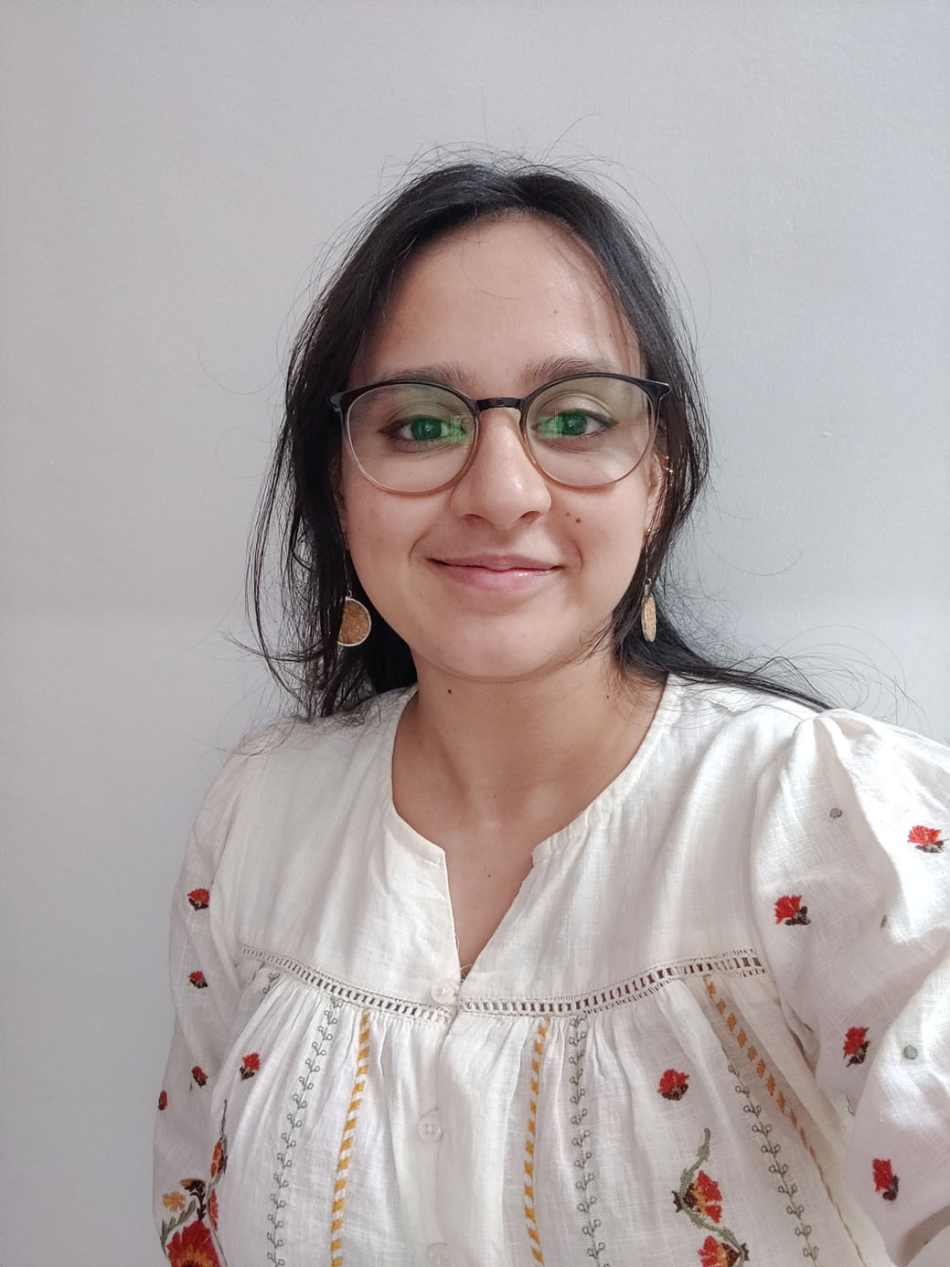
Radhika Malaviya

**Figure:**
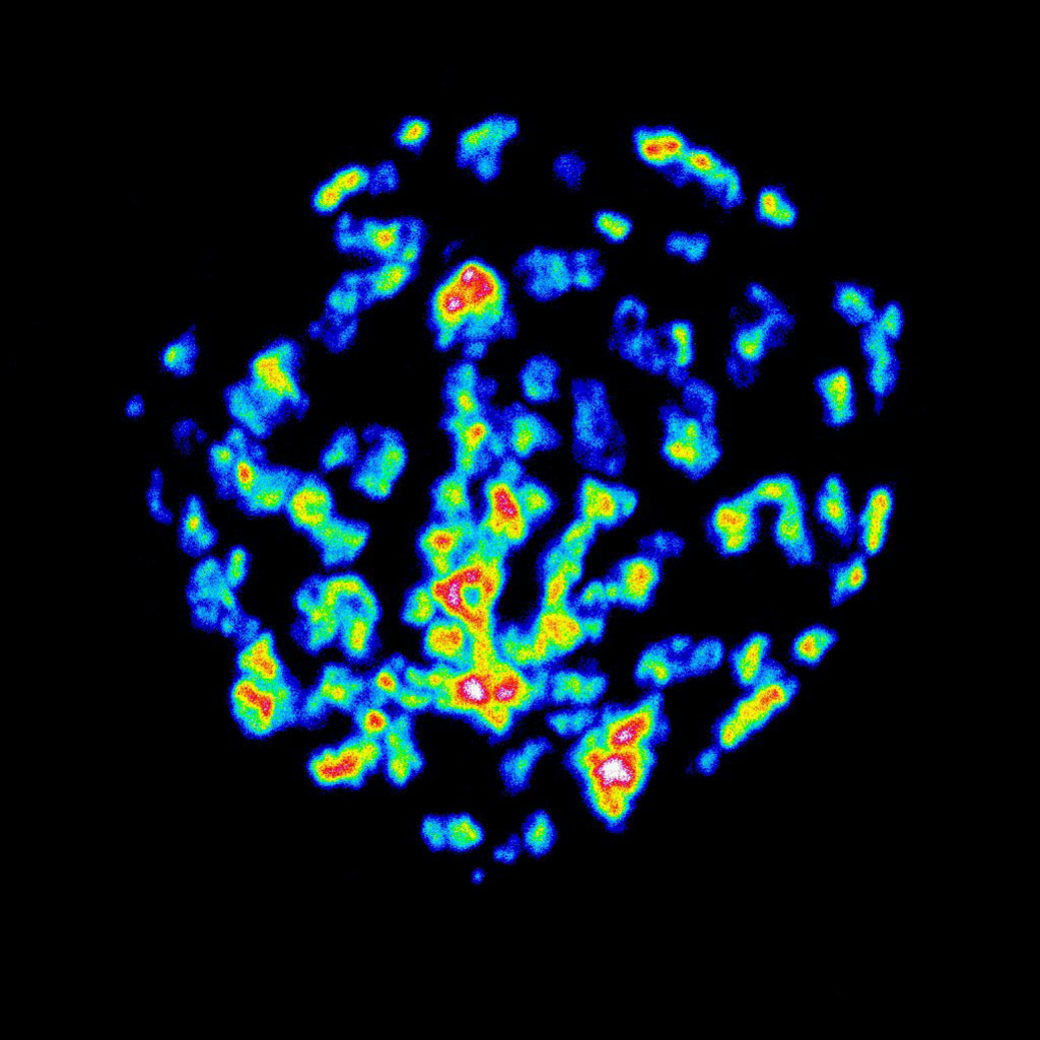
**MCF7 cells expressing ManII-GFP reveal a disorganized Golgi architecture under suspended conditions.** The image is represented in 16-colour mode.

**Which part of this research project was the most rewarding?**

**P.J.:** Golgi dynamics are inherently complex, and identifying a potential Golgi regulator from an extensive in silico screen was particularly challenging. One key finding was that targeting of a previously uncharacterized candidate – AXL – led to Golgi disruption in cancer cells, which was strikingly rescued in the presence of active Arf1 GTPase. This provided a solid foundation for the rest of our study. The most rewarding experience was having our work selected for oral presentations at several international conferences, including two Gordon Research Seminars, which was especially encouraging.

**A.S.:** One of the most rewarding parts of this project was resolving how a receptor like AXL could communicate with Arf1, which initially seemed quite puzzling. We spent considerable time exploring possible intermediate regulators, and, based on our analysis and prior knowledge, we focused on AMPK and its control of GBF1, a key activator of Arf1. It was particularly satisfying when our experiments showed that changes in AMPK activity affected GBF1 localization and Golgi organization, and that inhibiting AMPK could restore these defects. This moment brought the entire regulatory pathway together and was a key highlight of the study.

… curiosity drives the work, and each experiment – whether it succeeds or fails – contributes to growth

**What do you enjoy most about being an early-career researcher?**

**P.J.:** Being an early-career researcher allows me to pursue my ideas independently, while also giving me the freedom to seek guidance and collaborate across disciplines. I particularly value the wide range of possibilities this stage offers, to explore new directions, gain new learnings and shape my research career along the way.

**A.S.:** What I enjoy most about being an early-career researcher is the freedom to explore ideas, take intellectual risks and learn continuously. It's a stage where curiosity drives the work, and each experiment – whether it succeeds or fails – contributes to growth. I also appreciate that this phase allows space to question existing norms and push for improvements in academia, making it a more inclusive, supportive and sustainable environment for future scientists.

**R.M.:** As an early-career researcher, I really enjoy that I can continuously learn and take risks in my experiments. I appreciate the opportunity to integrate new ideas with existing methodologies to improve how we are doing science. This process has also challenged my preconceived notions and helped me grow further as a well-rounded researcher.

**What piece of advice would you give to the next generation of researchers?**

**P.J.:** I would advise the next generation to learn to enjoy the process of challenging themselves and pushing their boundaries. Take a step back occasionally to reflect on the bigger picture and stay aligned with your goals. Good research requires learning how to balance passion with patience. As my PhD mentor once told me, doing research is more like running a marathon than a 100 m sprint.

**A.S.:** I strongly resonate with the view expressed by Morten Meldal – that curiosity and a willingness to question are far more important than perfect grades. My advice to the next generation of researchers is to embrace curiosity and not shy away from challenging norms. Being a ‘rebel’ in this sense is not a weakness, but a strength – it drives critical thinking, encourages independent ideas and often leads to meaningful discoveries.

**R.M.:** As the next generation of researchers, we hold the power and duty to keep improving the field of science. I believe we, as a community, should constantly evaluate ourselves and our practices, keep what works well and improve areas that may be lacking. Keeping the spark of creativity and curiosity alive will always lead to good ideas and great science.

**What's next for you?**

**P.J.:** I have recently started as a postdoctoral associate at the Yale Cardiovascular Research Center. My current work focuses on how mechanical signals such as fluid shear stress regulate endothelial cell responses in the context of vascular biology and disease.

**A.S.:** After my PhD, I plan to pursue postdoctoral research in organelle and cancer mechanobiology to further deepen my technical expertise and address questions with translational potential. I am particularly interested in understanding the fundamental principles of signal transduction and how biochemical and mechanical signals are integrated within cells. I am currently actively seeking postdoctoral opportunities where I can apply the problem-solving skills developed during my PhD in a dynamic and interdisciplinary research environment.

**R.M.:** I am further investigating the role of AXL-Arf1 crosstalk in mechanosensing and Golgi organization, specifically in the context of normal versus cancer cells. After completing my PhD thesis, I would like to pursue postdoctoral research in organelle biology. I am specifically interested in looking at organelle dynamics in response to extracellular cues.
